# A Novel Method to Visualize the Dietary Macronutrient Composition of Smaller Visceral Fat Accumulation

**DOI:** 10.3389/fnut.2019.00194

**Published:** 2020-01-24

**Authors:** Tohru Yamaguchi, Naoki Ozato, Mitsuhiro Katashima, Kaori Sawada, Yoshihisa Katsuragi, Kazushige Ihara, Shigeyuki Nakaji

**Affiliations:** ^1^Health Care Food Research Laboratories, Kao Corporation, Tokyo, Japan; ^2^Department of Active Life Promotion Sciences, Graduate School of Medicine, Hirosaki University, Hirosaki, Japan; ^3^Department of Social Medicine, Graduate School of Medicine, Hirosaki University, Hirosaki, Japan

**Keywords:** visceral fat, dietary habit, macronutrients, ternary plot, compositional data, metabolic syndrome, visualization

## Abstract

The accumulation of visceral fat is considered a potential cause of a clustering of metabolic disorders including hypertension, hyperglycemia, and dyslipidemia. These disorders are some of the upstream determinants of serious diseases such as coronary heart disease, cerebrovascular disease, and dementia. In particular, the accumulation of visceral fat is considered to have a causal relationship with dietary habits. To clarify this relationship, we characterize dietary habits with dietary macronutrient composition and visceral fat accumulation with a measure of visceral fat area (VFA). We then employ a novel multiple regression model with VFA as the objective variable and macronutrient composition, gender, and age group as explanatory variables. The macronutrient composition is converted by the isometric log-ratio transformation since it is compositional data. The squared term of the transformed macronutrient composition is also included as an explanatory variable. To fit the data to the model, variable selection is performed based on Akaike's information criterion to exclude unnecessary interaction terms. The validity of the model is confirmed by a numerical simulation study. We then cross-sectionally analyze real-world data collected through community-wide health examinations of adults living in the Iwaki district in northern Japan. The macronutrient composition data is taken by the dietary history questionnaire and VFA is measured using a bioimpedance-type visceral fat meter. The main factors of macronutirent composition and their interactions with gender and age group are identified through analysis of variance and are significantly associated with VFA (*p* < 0.05). Moreover, the predicted VFA corresponding to the macronutrient composition stratified by gender and age group are obtained, and visualized seamlessly on a ternary plot. The results show that a diet with a high ratio of %protein to %fat generally corresponds to a lower VFA level. However, in middle-aged female subjects, higher VFA is found in lower %fat and higher %carbohydrate diets. In summary, the association between VFA and dietary macronutrient composition is significantly modulated depending on gender and age group in Iwaki district's adult population. The novel statistical analysis method in this study is useful in exploring favorable dietary macronutrient composition for lower level of visceral fat accumulation.

## Introduction

The accumulation of visceral fat is considered to be a potential cause of metabolic syndrome (1, 2), which is a clustering of metabolic disorders including hypertension, hyperglycemia, and dyslipidemia. Those disorders are some of the upstream determinants of serious diseases such as coronary heart disease, cerebrovascular disease, and dementia. A recent studies reported that subjects with abdominal obesity and a body mass index (BMI) within the normal range, have a higher risk of mortality compared to subjects without abdominal obesity (3–5). Therefore, visceral fat accumulation seems to be a significant indicator of individual health, regardless of BMI.

Although dietary habits and visceral fat accumulation are considered causally associated, the relationship is unclear because of the many other factors involved. Moreover, the relationship has significant inter- and intra-individual variety depending on life stage. In our study, we partially characterize dietary habits according to the dietary macronutrient energy composition of proteins (P), fats (F), and carbohydrates (C), collectively PFC, and analyze the effect on visceral fat accumulation, stratified by gender and age group.

The measurement of visceral fat accumulation can be challenging to introduce into large observational studies because it requires high-cost medical imaging technology such as X-ray computed tomography scanning or magnetic resonance imaging (6). In addition, it is nearly impossible to use statistical analysis to ascertain a reasonable relationship from the VFA and the PFC compositional data because of the inter-individual variety. The measurement problem is addressed by the introduction of the recently developed, low-cost bioimpedance-type visceral fat meter (7, 8), while the latter problem remains unsolved.

A multiple regression model with VFA as the objective variable and %P, %F, and %C as explanatory variables could have been considered to describe those relationship. However, the set of components %P, %F, and %C comprises compositional data do not fit the explanatory variables of the multiple regression model (9) because the individual variables do not follow the normal distribution and are highly correlated.

Although, compositional data is challenging in a regression model, as statistical modeling for compositional data has progressed since the 1980s (10), we can now use the isometric log-ratio transformation as an appropriate variable transformation method for compositional data (11, 12) (Appendix A) to overcome those non-normality and correlation problems. While the application of compositional data analysis can be found mainly in the field of geological science for the analysis of chemical composition of rocks (13–15), it has rarely been used in health and nutrition studies.

In this study, we introduce a multiple regression model with compositional regressor to clarify the relationship between macronutrient composition and visceral fat accumulation. The aim is to show the validity of the statistical model through a numerical simulation and demonstrate the usefulness of the model through an analysis of real-world data collected from Japanese adults.

## Materials and Methods

### Proposed Analysis Method

**Figure 1:**
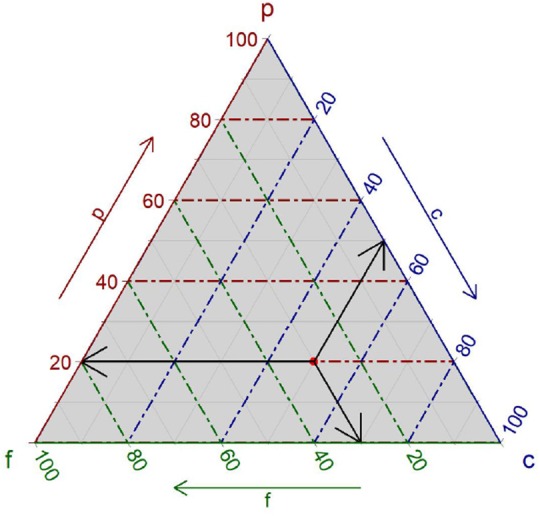
An example of ternary plots. A red point shows a compositional datum of P:F:C = 20:30:50. Three sub-compositions can be read at three axes showing by the cross-hair.

For compositional data with three components, we can use triangular, or ternary, plots. Ternary plots are similar to scatterplots but display a closed three-part sub-compositions (16). Figure 1 shows an interpretation of a ternary plot, For example, the data point of P:F:C=20:30:50 is highlighted on the ternary plot, and we can read the three sub-compositions from the three axes, making an equilateral triangle.

**Figure 2:**
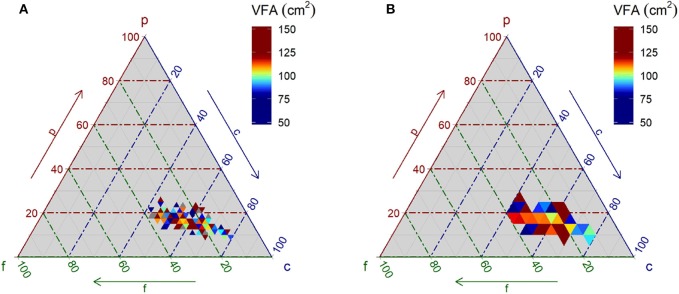
Relationship between macronutrient energy ratio and visceral fat area. Data of senior male subjects obtained through a questionnaire at community health examinations is plotted. The visceral fat area in color is the mean of data within each small, triangular area. The composition is discretized by 2.5% in **(A)** and 5% in **(B)**.

In a conventional, crude analysis without a statistical model, it is difficult to examine the relationship between PFC composition and VFA from a ternary plot. In Figure 2, the means of the data in the small triangle divided by (A) 2.5% and (B) 5% are plotted for real data. It is difficult to find a favorable PFC composition for a lower VFA level from the plots because the effect of dietary PFC composition on VFA is not deterministic due to considerable inter-individual varieties that are not explained by the PFC composition.

The proposed statistical model is a regression model with VFA as the objective variable and PFC composition as the explanatory variable. Here, the three components of PFC are converted into two components using the isometric log-ratio transformation, since it has only two degrees of freedom. When the first and second PFC components are determined, the third component is inevitably determined (Appendix A). We note that the regressor of PFC composition comprises continuous variables, even after the transformation. This implies a smooth VFA transition corresponding to the continuous transition of PFC composition.

### Numerical Simulation Study

A numerical simulation study is carried out to examine the validity of the model. The simulated VFA data and compositional PFC data are generated from a personal computer. The objective variable of VFA is converted by square root transformation to an approximately normal distribution. Moreover, the transformed PFC composition is too simple for the explanatory variable (16), thus, the inclusion of a second-order term of transformed PFC composition and its interaction is also considered. In the simulation study, the three statistical models used in the prediction of VFA are as follows.

Model 1: Explanatory variable of isometric log-ratio transformed PFC composition alone.

(1) $$\begin{array}{l} \sqrt{\text{VFA}}=\mu +\text{ilr}\left(\text{PFC}\right)+\varepsilon \\ \;\varepsilon \sim N\left(0,\sigma_{e}^{2}\right) \end{array}$$

Model 2: Transformed PFC composition and its squared term in the explanatory variables.

(2) $$\begin{array}{l} \sqrt{\text{VFA}}=\mu +\text{ilr}\left(\text{PFC}\right)+\text{sq}\left(\text{ilr}\left(\text{PFC}\right)\right)+\varepsilon \\ \;\varepsilon \sim N\left(0,\sigma_{e}^{2}\right) \end{array}$$

Model 3: PFC, square term of PFC, and their interaction term.

(3) $$\begin{array}{l} \sqrt{\text{VFA}}=\mu +\text{ilr}\left(\text{PFC}\right)+\text{sq}\left(\text{ilr}\left(\text{PFC}\right)\right)+\text{ilr}\left(\text{PFC}\right)\\ \;\times \text{sq}\left(\text{ilr}\left(\text{PFC}\right)\right)+\varepsilon \\ \;\varepsilon \sim N\left(0,\sigma_{e}^{2}\right) \end{array}$$

Here, μ is an overall mean and ε is an error term following normal distribution, with the mean as 0 and variance as $\sigma_{e}^{2}$. $\sqrt{\text{VFA}}$ represents the square root-transformed VFA, and ilr(**PFC**) represents a vector of PFC composition converted by the isometric log-ratio transformation. Moreover, the sq(ilr(**PFC**)) represents the squared term of the converted PFC composition. In the simulation study, we define a region of interest (ROI) as a 95% region of dietary PFC composition from health examination data of senior male subjects. The experimental procedure is as follows.

1. Generate one random PFC compositional datum from compositional normal distribution where the mean and variance are calculated from the health examination data of senior male subjects.
2. Accept as a datum if it is in the ROI.
3. Repeat procedures 1 and 2 until the number of accepted data reaches 300.
4. Generate 300 random VFA data from a normal distribution with the mean as 10 and the standard deviation as 0.5, and square them (the mean of squared values is nearly 100).
5. Add an artificial bias of 10 cm^2^/10% in the slope to the VFA data according to designed patterns.
6. Fit the data to Model 1, Model 2, and Model 3.
7. Predict VFA distributions using the fitted models by 1% intervals of %P, %F, and %C.
8. Visualize the predicted distribution on a ternary plot limited to ROI.
9. Evaluate reproducibility by comparing the plots with the original designed pattern.

**Figure 3:**
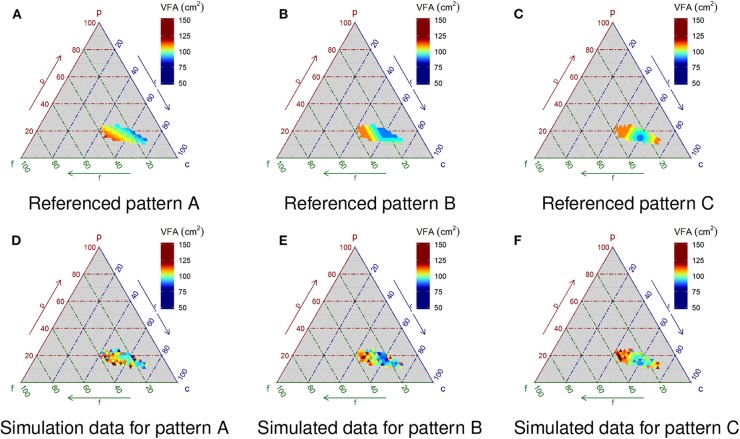
Simulated pattern of VFA and simulated data. Pattern A is defined as a higher %P to %F ratio, decreasing VFA. Pattern B is defined as <27.5%F and more than 12.5%P, meaning visceral fat does not accumulate. Pattern C is defined by an optimum point for lower VFA in terms of %P:%F:%C as15%:25%:60%, respectively. **(D–F)** Are examples of simulated data with Gaussian noise corresponding to **(A–C)**, respectively.

The three patterns of the VFA modulated by PFC composition are studied (Figures 3A–C). Pattern A is defined as a higher %P to %F ratio, decreasing VFA. Pattern B is defined as <27.5%F and more than 12.5%P, meaning visceral fat does not accumulate. Pattern C is defined by an optimum point for lower VFA in terms of %P:%F:%C as 15%:25%:60%, respectively. Figures 3D–F are examples of simulated data that add artificial noise in patterns A, B, and C, as shown in Figures 3A–C, respectively.

### Real-World Data Study

Following the simulation study, we examine the usefulness of the statistical model using real-world data. The subjects are residents over 20 from the Iwaki district of Hirosaki city in northern Japan who had taken part in annual free health examinations after they have provided written informed consents. Based on the resident registration, all of adult residents are invited. About 10% of the 11,000 adult residents of the Iwaki district have voluntarily taken part in the examination each year. For this study, subjects who had undergone at least one examination in 2015, 2016, or 2017 were suitable for analysis. The latest dataset was analyzed for those who had undergone examinations in multiple years. After eliminating subjects with incomplete VFA and nutrition data, 1,538 respondents remained.

The dietary survey was conducted using the validated “brief-type self-administrated diet history questionnaire” (BDHQ) (17, 18). The BDHQ, which listed 80 questions on intake frequency of foods and beverages over the last one month, was sent to subjects by mail before the annual health examination. Individuals were asked to complete the questionnaire beforehand and bring it with them on the day of the health examination. Trained investigators carefully checked for missing values when receiving the questionnaires. The checkmarks and numerical figures on the completed questionnaires were captured and entered with an optical character reader, therefore missing value could be checked simultaneously. Nutritional data was computed with personal computers using a predefined algorithm developed using *Standrad Tables of Food Compositon in Japan* (19). The missing value for the food frequency question is considered to be “not eaten.”

To calculate the macronutrient energy composition from weight intakes, we used the Atwater system, that is, protein and carbohydrate intakes were multiplied by 4 kcal/g and fat intake was multiplied by 9 kcal/g. The calculations excluded six female subjects whose average energy intake was <600 kcal/day because this amount is not physiologically sufficient to sustain life (20).

The accumulation of visceral fat was assessed as a square measure of visceral fat using a bioimpedance type visceral fat meter (EW-FA90, Panasonic Corp., Osaka, Japan). The device has four electrodes on a belt and was placed at a subject's umbilical level while the subject was in a standing position (7). To measure VFA, the device injected a weak alternating electric current through two electrodes placed near the umbilici and the back. The induced electric voltage between the two electrodes placed on the right lateral abdominal area was measured. Because the injected current was controlled as constant, the induced voltage was equivalent to a bioimpedance of the deep inner abdomen where visceral fat might be accumulated. Simultaneously, much like a measuring tape, the device measured the waist circumference using the length of the extended belt.

From the bioimpedance, the waist circumference, and the subject's sex, the device derived the VFA using a built-in pre-defined algorithm. The validity of the device was confirmed by a high correlation coefficient with measurement by an X-ray computed tomography scan (7). It has also been approved as a medical device by the Japanese government. A trained registered nurse or clinical laboratory technician conducted the measurements in accordance with standard operating procedures. In the cases of eight female and six male subjects with measurement errors due to VFA levels of <10 cm^2^, the VFA was treated as 10 cm^2^.

Behavioral data was collected through the self-reported questionnaire prepared specifically for the community health examination. The questionnaire surveyed a wide range of behavioral habits and health conditions, including dietary habits, exercise habits, sleeping habits and use of medications. A portion of these data was presented as the subject's background information.

### Statistical Analysis

The subjects were stratified by age for both genders. Respondents aged 20 to 34 were defined as “young,” those 35 to 59 as “middle-aged,” and those 60 or older as “senior.” Age 35 was set as a boundary because visceral fat begins to accumulate at approximately this age in the general Japanese population (21). Age 60 was set as a boundary for seniors because people typically begin to retire from full-time work at this age, and most women have reached menopause. The anthropometric continuous variables were summarized with the quartile as well as the mean and standard deviation. The statistical method used in the real-world study is based on the method used in the simulation study however, categorical data of GENDER and AGEGROUP were included as main-factor and interaction terms.

The significance level was set to *p* = 0.05 (two tails) in all statistical tests. The results were considered statistically significant when the *p*-value was smaller than the significance level. We used R statistical software and environment (version 3.6.0) and the extension packages of “composition” (16) and “ggtern” (22) concurrently to treat and visualize the compositional data.

## Results

### Numerical Simulation Study

**Figure 4:**
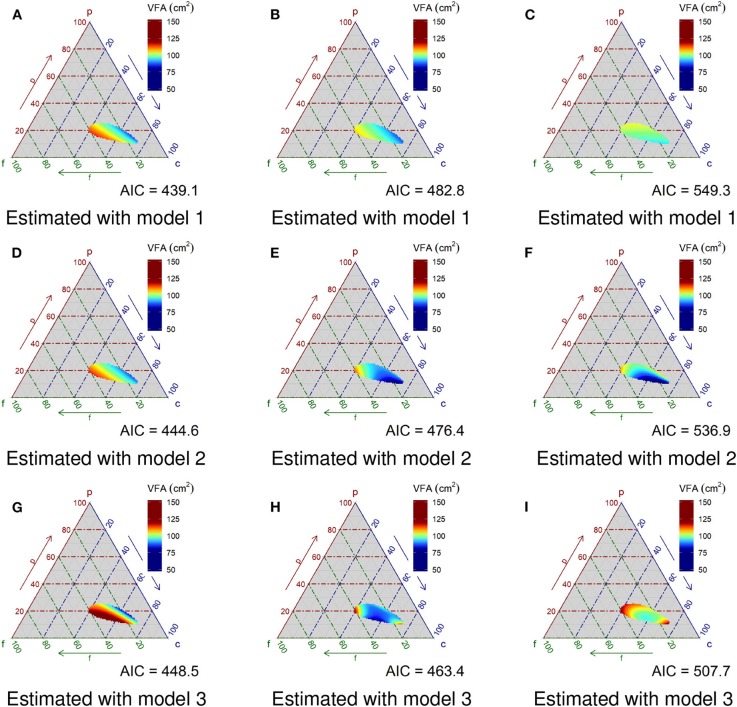
Evaluation for reproducibility of the statistical model. **(A–C)** Show reproduced visceral fat areas with Model 1 for patterns A, B, and C, respectively. **(D–F)** Are those with Model 2, and panels **(G–I)** are those with Model 3. Model 1 has the transformed compositional PFC data as an explanatory variable. Model 2 has the square term of the compositional PFC in addition to Model 1. Model 3 has the interaction terms of compositional PFC and its square term in addition to Model 2.

The results of the simulation study (*n* = 300) are shown in Figure 4. With respect to Pattern A (Figure 3A), the estimation under Model 1 (Figure 4A), which is the simplest model, most effectively reproduces the original pattern. By contrast, the reproduced distribution using Model 3 (Figure 4G) is the poorest compared against both Models 1 and 2 (Figures 4A,D). Applying Akaike's information criterion (AIC), Model 1 yields the lowest estimate and thus is the best relative to the three models (AIC estimates for Models 1, 2 and 3: 439.1, 444.6 and 448.5, respectively). In terms of the visual observation of the ternary plots for both Patterns B and C (Figures 3B,C, respectively), applying the most complex Model 3 (Figures 4H,I, respectively) appears best, followed by Model 2 (Figures 4E,F, respectively), which, in turn, exceeds Model 1 (Figure 4B). The AIC estimates for Model 3 generated the lowest values (i.e., 463.4 and 507.7 for Patterns B and C, respectively), confirming Model 3 is the best option for Patterns B and C.

### Real-World Data Study

The characteristics of the subjects are shown in Table 1 for females and Table 2 for males. The data of 932 females, excluding 6 females with unusual energy intake, and 600 males are analyzed. The proportion of farmers among the young, middle-aged, and senior males is 34, 33, and 50%, respectively, much higher than that of the total Japanese adult male population, which is ~4%.

**Table 1:** Background descriptive statistics by age-group in female subjects.

	**N**	**Young**				**Middle-aged**				**Senior**			
		*****N*** = 131**				*****N*** = 391**				*****N*** = 410**			
Age (y)	932	26.0^a^	30.0^b^	33.0^c^	(29.3 ± 4.3)	42.0^a^	49.0^b^	55.0^c^	(48.6 ± 7.4)	65.0^a^	68.0^b^	75.0^c^	(69.9 ± 6.3)
Weight (kg)	932	47.0^a^	51.4^b^	57.0^c^	(53.2 ± 9.4)	49.0^a^	54.0^b^	61.0^c^	(55.8 ± 9.7)	47.2^a^	52.5^b^	57.9^c^	(53.1 ± 8.4)
BMI (kg/m^2^)	932	18.3^a^	19.9^b^	22.3^c^	(20.8 ± 3.8)	19.6^a^	21.7^b^	24.2^c^	(22.3 ± 3.6)	21.0^a^	22.9^b^	25.0^c^	(23.2 ± 3.4)
VFA (cm^2^)	932	26^a^	37^b^	57^c^	(46 ±31)	38^a^	58^b^	82^c^	(64 ±33)	54^a^	73^b^	94^c^	(75 ±32)
Energy (kJ)	932	5201^a^	6326^b^	7849^c^	(6575 ± 1870)	5578^a^	6695^b^	7869^c^	(6874 ± 1911)	5941^a^	7112^b^	8486^c^	(7369 ± 2292)
%Protein	932	13.8^a^	14.8^b^	17.1^c^	(15.4 ± 2.5)	14.3^a^	15.8^b^	17.6^c^	(16.0 ± 2.6)	14.9^a^	16.6^b^	18.9^c^	(16.9 ± 3.1)
%Fat	932	26.3^a^	30.3^b^	33.6^c^	(30.1 ± 5.5)	25.3^a^	28.4^b^	32.1^c^	(28.6 ± 5.5)	23.2^a^	26.9^b^	30.3^c^	(26.7 ± 5.5)
%Carbohydrate	932	51.0^a^	54.4^b^	58.6^c^	(54.6 ± 7.0)	50.7^a^	55.7^b^	59.9^c^	(55.4 ± 7.2)	51.3^a^	55.4^b^	61.5^c^	(56.3 ± 7.7)
Farmer: Yes	932	8.4% (11)				25.6% (100)				39.8% (163)			
Exercise: Yes	932	59% (77)				62% (243)				48% (198)			
Smoking: Yes	931	13.7% (18)				13.6% (53)				2.7% (11)			
No		73.3% (96)				68.7% (268)				89.8% (368)			
Previous		13.0% (17)				17.7% (69)				7.6% (31)			
Alchol: Yes	932	32.1% (42)				40.2% (157)				19.0% (78)			
No		62.6% (82)				55.2% (216)				78.0% (320)			
Previous		5.3% (7)				4.6% (18)				2.9% (12)			

*a, b, and c represent the lower quartile a, the median b, and the upper quartile c for continuous variables. x±s represents $\bar{X}\pm 1$ SD. Numbers after percents are frequencies. Young, Middle-aged, and Senior are defined as 20 to 34 years old, 35 to 59 years old, and 60 and older, respectively. BMI, body mass index; VFA, visceral fat area*.

**Table 2:** Background descriptive statistics by age-group in male subjects.

	**N**	**Young**				**Middle-aged**				**Senior**			
		*****N*** = 98**				*****N*** = 278**				*****N*** = 224**			
Age (y)	600	27.0^a^	30.5^b^	34.0^c^	(29.8 ± 4.4)	40.0^a^	47.0^b^	54.0^c^	(47.2 ± 7.5)	65.0^a^	68.0^b^	75.0^c^	(70.3 ± 6.7)
Weight (kg)	600	61^a^	68^b^	74^c^	(71 ±14)	64^a^	68^b^	75^c^	(70 ±11)	57^a^	63^b^	70^c^	(64 ± 9)
BMI (kg/m^2^)	599	20.4^a^	22.9^b^	24.9^c^	(23.6 ± 4.4)	21.7^a^	23.7^b^	25.8^c^	(24.0 ± 3.3)	21.8^a^	23.5^b^	25.8^c^	(23.8 ± 2.9)
VFA (cm^2^)	600	54^a^	82^b^	122^c^	(93 ± 58)	79^a^	104^b^	136^c^	(109 ± 45)	82^a^	112^b^	146^c^	(114 ± 46)
Energy (kJ)	600	6854^a^	8263^b^	9544^c^	(8393 ± 2397)	7283^a^	8586^b^	10318^c^	(8899 ± 2351)	7662^a^	9061^b^	10939^c^	(9481 ± 2636)
%Protein	600	12.9^a^	14.8^b^	16.5^c^	(15.1 ± 2.9)	13.2^a^	14.6^b^	16.2^c^	(14.9 ± 2.4)	14.0^a^	15.9^b^	17.8^c^	(16.2 ± 2.9)
%Fat	600	22.9^a^	26.5^b^	30.6^c^	(26.6 ± 5.8)	21.2^a^	24.8^b^	28.6^c^	(25.0 ± 5.5)	21.6^a^	25.6^b^	28.5^c^	(25.1 ± 5.2)
%Carbohydrate	600	53.0^a^	58.1^b^	64.3^c^	(58.3 ± 7.9)	55.7^a^	60.0^b^	64.9^c^	(60.0 ± 7.2)	54.0^a^	58.3^b^	63.3^c^	(58.7 ± 7.2)
Farmer: Yes	600	34% (33)				33% (91)				50% (111)			
Exercise: Yes	600	51% (50)				64% (177)				52% (116)			
Smoking: Yes	600	41% (40)				36% (99)				16% (35)			
No		47% (46)				27% (76)				49% (110)			
Previous		12% (12)				37% (103)				35% (79)			
Alchol: Yes	600	51.0% (50)				73.0% (203)				68.3% (153)			
No		45.9% (45)				24.5% (68)				19.6% (44)			
Previous		3.1% (3)				2.5% (7)				12.1% (27)			

*a, b, and c represent the lower quartile a, the median b, and the upper quartile c for continuous variables. x±s represents $\bar{X}\pm 1$ SD. Numbers after percents are frequencies. Young, Middle-aged, and Senior are defined as 20 to 34 years old, 35 to 59 years old, and 60 and older, respectively. BMI, body mass index; VFA, visceral fat area*.

The median VFA in middle-aged males is above 100 cm^2^, which the Japanese Society for the Study of Obesity (23) defines as the threshold level for visceral fat obesity. The median VFA is higher for senior males than that for middle-aged males, although the median BMI is almost the same. The VFA in female subjects, though, is in the normal range, even in 75th percentile of all age groups. However, the median VFA of senior females is higher than that of middle-aged females, showing the same trend observed in males. In the statistical analysis, we use the modified statistical model adding the terms of gender and age group from the model used in the simulation study. First, variable selection is performed based on minimization of the AIC. The selection begins with the main factors forced on the model. After that, the best combination of explanatory variables is selected from all possible regressions of the interaction terms. The final model is shown in Equation (4) as:

(4) $$\begin{array}{l} \sqrt{\text{VFA}}=\mu +\text{ilr}\left(\text{PFC}\right)+\text{sq}\left(\text{ilr}\left(\text{PFC}\right)\right)+\text{GENDER}\\ \;+\text{AGEG}+\text{ilr}\left(\text{PFC}\right)\times \text{GENDER}+\text{ilr}\left(\text{PFC}\right)\times \text{AGEG}\\ \;+\text{GENDER}\times \text{AGEG}+\varepsilon \\ \;\varepsilon \sim N\left(0,\sigma_{e}^{2}\right) \end{array}$$

where GENDER and AGEG are categorical data of gender and age group, respectively.

The result of the analysis of variance (ANOVA) table, in which all of the subject data is fitted on the final model expressed in Equation (4), is shown in Table 3. The main effects of PFC composition, gender, and age group are statistically significant (*p* < 0.05). Moreover, the interaction of PFC and gender, and PFC and age group are significant, implying that the effect of PFC composition on VFA is different depending on gender and age group. The contributing ratio of each term is presented in the %Contribution column in Table 3. The cumulative contribution ratio, or determination coefficient for the model, is 0.26, implying that these explanatory variables are not deterministic for VFA.

**Table 3:** Analysis of variance table for visceral fat area.

	**Df**	**Sum Sq**	**Mean Sq**	***F*-value**	**p-value**	**%Contribution**
ilr(PFC)	2	169.03	84.51	18.31	<0.001	1.79
sq(ilr(PFC))	3	15.18	5.06	1.10	0.350	0.16
GENDER	1	1723.41	1723.41	373.33	<0.001	18.20
AGEG	2	433.22	216.61	46.92	<0.001	4.58
ilr(PFC):GENDER	2	63.82	31.91	6.91	0.001	0.67
ilr(PFC):AGEG	4	50.51	12.63	2.74	0.028	0.53
GENDER:AGEG	2	19.28	9.64	2.09	0.124	0.20
Residuals	1515	6993.68	4.62			73.87

*Df, degree of freedom; Sum Sq, sum of squares; Mean Sq, mean squares*.

**Figure 5:**
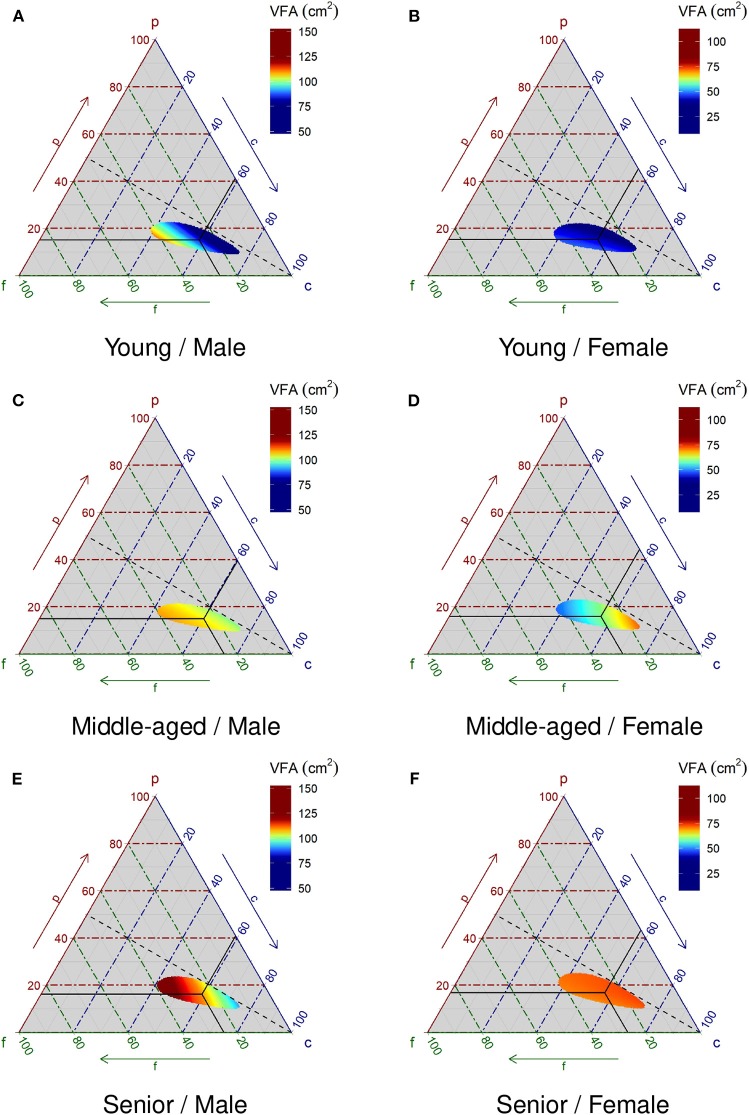
Relationship between macronutrient composition and visceral fat area. The visceral fat area in color is predicted by the statistical model in Equation (4). The statistical model is a multiple regression model with the square root converted visceral fat area as the objective variable and the isometric log-ratio converted macronutrient energy ratio and its squared term, gender, age group, and those interaction terms as explanatory variables. The cross-hair indicates the center of the compositional data in each stratum. The dotted line indicates equal ratio of protein and fat. **(A)**, **(C)**, and **(E)** are male groups of Young, Middle-aged, and Senior, respectively. **(B)**, **(D)**, and **(F)** are female groups of Young, Middle-aged, and Senior, respectively. Young, Middle-aged, and Senior are defined as 20 to 34 years old, 35 to 59 years old, and 60 and older, respectively. Scales for males and females are different.

Finally, the predicted value of VFA stratified by gender and age group is shown in ternary plots in Figure 5. The VFA is expressed seamlessly in color. The crosshairs in the figures are the center of the data in each stratum and the dotted line shows the ratio of %P to %F as 1. The color scales differ by gender because the level of VFA in females is much lower than that in males.

## Discussion

In this study, a regression model with compositional regressor is proposed to explore the relationship between dietary PFC composition and VFA. The novelties of the model are as follows: (1) the conversion of the PFC composition by isometric log-ratio transformation as a treatment for the compositional data; and (2) the inclusion of the squared term of the converted PFC as explanatory variables. The validity of the model is examined in the numerical simulation study. The best model depending on the underlying complexity of the data distribution against PFC composition should be determined by the variable selection based on the AIC. Variable selection is therefore performed by fitting real-world data, and the moderately complex model is selected.

Comparing the ternary plots in Figures 2A,B with Figure 5E, although all plots are derived from the same raw data, Figure 5E depicts much clearer evidence of the relationship between VFA and PFC composition than patterns depicted in Figures 2A,B. This demonstrates the strength of the proposed statistical model.

For young males, the transition of VFA on the ternary plot is along the projection line where the %P to %F ratio equals 1. Thus, subjects consume a diet with a low ratio of %P to %F and tend to have high VFA levels. On the other hand, for young females, the effect of dietary PFC composition is small and the level of VFA is much lower than that of males. For middle-aged males, the subjects consume lower ratios of %P to %F and tend to have higher VFA levels, much like young males. Middle-aged females consume lower %F and high %C, and tend to have higher VFA levels. Senior males consume a higher %F and tend to have higher VFA levels. On the other hand, for senior females, the effect of dietary PFC composition on VFA levels is not clear.

The relationship between a higher ratio of %P to %F and lower VFA observed in young and middle-aged males is consistent with another study of the Japanese population (21). Moreover, studies indicating that Canadian subjects with a high protein diet have less body fat (24) and that European and Brazilian subjects in six studies with a high-protein diet have higher energy expenditure (25) are also consistent with our results. On another front, our study indicates that a lower carbohydrate diet tends to result in lower VFA levels among middle-aged females. This result is consistent with the result of Shai et al.'s randomized controlled trial, which found that a low carbohydrate diet is effective in maintaining body weight (26).

There are several studies on different dietary macronutrient compositions (26–30). In these randomized controlled trials, the comparison of one condition to another is made in a limited subject group. The conclusions from these studies are somewhat fragmented. In contrast, our study seamlessly visualizes the relationship between PFC composition and VFA stratified by gender and age groups, therefore allowing for an omnibus interpretation of the relationship.

One of the limitations of this study is that we studied only one population to fit the statistical model. In other populations, either in Japan or in foreign countries, another statistical model might be selected, producing very interesting results. Furthermore, in this study, we did not focus on the quality of P, F and C individually. Specifically, we did not consider the source of protein, animal vs. plant (31); the type of fat, saturated vs. unsaturated (32), and the quality of carbohydrate, low glycemic index vs. high glycemic index (33–35). These qualitative aspects have been shown to be of utmost importance in their link to metabolic disorders.

We used community health examination data in the Iwaki district as a motivating example. The BDHQ we used as a food frequency questionnaire has not validated for this population. This is another one of the study's limitations. The residents of Aomori Prefecture, which includes the Iwaki district, have the shortest lifespan of Japan's 47 prefectures (36) because of unhealthy dietary habits such as a limited variety of foods at each meal, overeating, and high salt and junk-food intake, as well as high rates of smoking, alcohol consumption, and limited exercise habits. In the middle-aged males, the median VFA is above 100 cm^2^, indicating that more than half the subjects may have metabolic syndrome, even though the median BMI for the group is below 25 kg/m^2^, which is in the normal range (Table 2). Therefore, these results should only be generalized with care to the broader Japanese population.

## Conclusion

We introduce a novel regression model composed of PFC compositional data as the explanatory variable, and clearly visualize the relationship between dietary macronutrient composition and VFA to convincingly overcome inter-individual variability. The ANOVA results clearly show that dietary macronutrient composition and its interaction with gender and age group are significantly associated with VFA (*p* < 0.05). Visceral fat accumulation is therefore modulated differently by dietary macronutrient composition according to gender and age group. More specifically, a diet with a high ratio of %P to %F is generally suitable for the prevention of visceral fat accumulation. However, a diet with low %F and high %C should be avoided for middle-aged females, or at least for those of the Iwaki district. One of the main limitations of this study is that only one population was applied to the statistical model. The comparison of statistical models for other populations in Japan or in foreign countries would be very interesting for further investigation. Also, a statistical model taking into account the quality of protein, fat and carbohydrates would also be worthwhile for further study. In summary, the statistical model presented in this paper is useful in exploring the relationship between dietary macronutrient composition and visceral fat accumulation.

The authors expect to employ the model in future studies.

## Data Availability Statement

The raw data supporting the conclusions of this article will be made available by the authors on reasonable request to any qualified researcher.

## Ethics Statement

The ethical committee of the Graduate School of Medicine, Hirosaki University, approved the questionnaire and data collection. We respected the spirit of the Declaration of Helsinki and obtained documented informed consent from subjects after detailed explanations. The data analysis was carried out after the approval of the data-management committee in the COI Research Initiatives Organization, Hirosaki University.

## Author Contributions

TY performed the data analysis and prepared the manuscript. All authors contributed to the study design and interpretation of results, design, and execution of the community health examination and the data collection, read and approved the final manuscript.

### Conflict of Interest

The authors declare that this study received funding from Kao Corporation (Tokyo, Japan). The funder was involved in the study design, data collection and analysis, decision to publish, and preparation of the manuscript. TY, NO, MK, and YK were employed by Kao Corporation. The remaining authors declare that the research was conducted in the absence of any commercial or financial relationships that could be construed as a potential conflict of interest.

## Abbreviations

%C: energy percentage of carbohydrate
%F: energy percentage of fat
%P: energy percentage of protein
ANOVA: analysis of variance
BDHQ: brief type self-administrated diet history questionnaire
P: protein
F: fat
C: carbohydrate
PFC: a set of protein, fat, and carbohydrate composition
VFA: visceral fat area.

## Appendix A, The Isometric Log-Ratio Transformation

As the sum of %P, %F, and %C is always equal to 100%, the PFC data is a kind of compositional data with 2° of freedom, but with three components. The characteristics of compositional data are: 1) each component does not follow the normal distribution and 2) components strongly correlate with each other. These characteristics are unfavorable as explanatory variables of multiple regression models – they need appropriate variable transformation. Therefore, we employed the isometric log-ratio transformation (12, 37). Specifically, for the three components of compositional data, %P, %F, and %C, the isometric log-ratio transformation ilr(**PFC**) is expressed as:

(A-1) $$\begin{array}{l} \text{ilr}\left(\left[\begin{array}{c} \%\text{P}\\ \%\text{F}\\ \%\text{C} \end{array}\right]\right)=\left[\begin{array}{c} \sqrt{\frac{1}{2}}\text{log}\frac{\%\text{F}}{\%\text{P}}\\ \sqrt{\frac{2}{3}}\text{log}\frac{\%\text{C}}{\sqrt{\%\text{P}\%\text{F}}} \end{array}\right] \end{array}$$

Thus, those three components can be converted into independent two components that almost follow the normal distribution.
